# Clinical profile and treatment outcome of acute intussusception among children in eastern Ethiopia: A seven years retrospective study

**DOI:** 10.3389/fped.2022.968072

**Published:** 2022-11-28

**Authors:** Dawit Desta Tesfaye, Burka Mohammed Adem, Indeshaw Ketema, Ame Mehadi, Bajrond Eshetu, Tilahun Teshager, Henock Asfaw, Assefa Desalew

**Affiliations:** ^1^ School of Medicine, College of Health and Medical Sciences, Haramaya University, Harar, Ethiopia; ^2^ School of Nursing and Midwifery, College of Health and Medical Sciences, Haramaya University, Harar, Ethiopia

**Keywords:** acute intussusception, treatment outcome, pediatrics, harar, eastern Ethiopia

## Abstract

**Background:**

Acute intussusception is the main cause of abdominal surgical emergencies worldwide in young children, with an incidence of approximately 1 to 4 per 2,000 children. An accurate estimate of the treatment outcomes of acute intussusception in children is unknown in low-and middle-income countries like Ethiopia. Hence, this study aimed to determine the clinical profile, treatment outcomes of acute intussusception and its associated factors among children admitted to Hiwot Fana Specialized University Hospital in eastern Ethiopia.

**Methods:**

An institutional-based retrospective cross-sectional study was conducted from November 01 to 30, 2021, among children admitted and managed for acute intussusception. All medical records of children admitted and managed for acute intussusception at Hiwot Fana Specialized University hospital between January 2014 and December 2020 were included. Data were collected using pretested structured checklists through a review of medical records, entered and analyzed using Statistical Package for Social Sciences version 25.0. Chi-square (*χ*^2^) tests were applied to determine the associated factors with treatment outcome. The statistical significance was considered at a *p*-value < 0.05.

**Results:**

In this chart review of children, 13.3% (95% CL: 11.8–14.8) died. The median age of the study participant was 13 months. The majority, 72% were male and 76% were less than 24 months old. Regarding the clinical profile; abdominal pain (94.7%), vomiting (93.3%), bloody diarrhea (70.7%), and abdominal distention (76.0%) were the most common clinical presentations. Age less than 24 months [*X*^2 ^= 8.13 (df = 1); *p* = 0.004], preoperative vital signs [*X*^2 ^= 19.21 (df = 2); *p* = 0.000], intraoperative findings [*X*^2 ^= 18.89 (df = 1); *p* = 0.000], and postoperative complications [*X*^2 ^= 14.60 (df = 1); *p* = 0.000] were significantly associated with treatment outcome of acute intussusception.

**Conclusion:**

In this chart review, the overall mortality rate in children was relatively high. One in seven children died from acute intussusception. Age less than 24 months, preoperative vital signs, intraoperative findings, and postoperative complications were significantly associated with acute intussusception treatment outcomes. Surgical management was the only treatment performed in all cases. Delayed presentation of patients and lack of other treatment modalities such as non-surgical interventions are serious concerns in this facility. The initiation of non-surgical reduction may reduce the need for surgical intervention-related complications, and child mortality.

## Introduction

Acute intussusception is the main cause of abdominal surgical emergencies such as acute intestinal obstruction and abdominal pain worldwide in the paediatric population (1, 2). Intussusception happens when one segment of the intestine invaginates into the lower segment of the intestinal wall. When the intussuscepted segment propagates distally with the intestine, it also draws with the blood vessels that compromise circulation. If intussusception is not timely diagnosed and properly reduced, intestinal tissue infarction and perforation may happen and cause child mortality (1, 2). Approximately 74 intussusceptions occur annually per 100,000 infants worldwide (2). It was first described in 1674 in the younger population, it may be caused by unknown aetiology in 75% of cases (3, 4). However, 25% of cases might be caused by underlying conditions such as Meckel's diverticulum, polyp, rheumatoid purpura, and lymphoma (5, 6).

The majority of intussusception cases are classified as primary or idiopathic. The pathological causes of intussusception predominate over the age of 3 years, with Meckel's diverticulum, intestinal duplication, polyps, and intestinal malignancy being the most common lead points (6–8). In some children with acute intussusception, there is the possibility of spontaneous reduction and recovery (1). However, in cases of persistent acute intussusception, which may result in circulatory compromise and subsequently bowel necrosis, requires surgical intervention. Untreated acute intussusception is a potentially life-threatening condition. So, early diagnosis, and management with appropriate surgical or non-surgical reduction have resulted in a significant reduction in morbidity and mortality (1).

Clinically, younger children who suffered from acute intussusception may present with a variety of signs and symptoms such as vomiting, abdominal distension, colicky abdominal pain, and bloody stools after a delay in presentation (9). Moreover, to confirm the diagnosis, advanced diagnostics imaging studies may be needed such as abdominal radiography, ultrasonography, barium studies, and computed tomography in particular cases (10, 11).

The management of acute intussusception is either surgical or non-surgical interventions such as hydrostatic and pneumatic reduction under fluoroscopy or ultrasound guidance is currently the recommended treatment modality (7, 12, 13). Factors that are linked with failure of non-surgical intervention are the duration of symptoms **>**48 h, hematochezia, abdominal distension, presence of complications identified on ultrasound, and unsuccessful hydrostatic or pneumatic reduction (12, 13).

In many low-income countries, including Ethiopia, the management of acute intussusception is exclusively surgical intervention and involves manual reduction or resection of a necrotized or perforated bowel. The management of uncomplicated cases with hydrostatic reduction is not well-practiced in low-income countries (14).

The case fatality rate of intussusception was higher in African countries (9%) than in other regions of the world (<1%) (2). Despite the high prevalence of acute intussusception in sub-Saharan African countries, little is known about the clinical presentation and management outcomes of acute intussusception in the pediatric population (15). Therefore, this study aimed to determine the treatment outcomes of acute intussusception and its associated factors among children in eastern Ethiopia.

## Methods and materials

### Study setting and period

The study was conducted from November 01 to 30, 2021 in the Hiwot Fana Specialized University Hospital (HFSUH), eastern Ethiopia, among children diagnosed and treated for acute intussusception between January 2014 and December 2020. The hospital is located 526 km east of Addis Ababa, the capital city of Ethiopia, in Harar town, the capital city of Harari Regional State and East Hararghe Zone of Oromia Regional State. Harar town has two public hospitals (Jugol General Hospital and HFSUH), one Federal Police Hospital, one private General hospital, four public health centers, and one Family Guidance Association.

HFSUH is serving as a teaching institution for health science students from different Health Science Colleges and Universities in eastern Ethiopia under the umbrella of Haramaya University. It provides 24-h comprehensive services for more than 5.8 million populations with different demographic and socioeconomic backgrounds from the entire surrounding of the Eastern part of the country including the Harari Regional State, Dire Dawa City Administration, Eastern part of Oromia Regional State (Eastern and Western Hararghe Zones) and the Somali Regional State. The hospital provides emergency medicine, internal medicine, neurology, general surgery, orthopedics, neurosurgery, obstetrics and gynecology, pediatrics, radiology, dermatology, pathology, oncology, anesthesiology, and neonatal care specialty services for the entire population from eastern Ethiopia. The department of Pediatrics has six units including Pediatric Ward, PICU, NRU, NICU, OPD, and Chronic Follow-up Units.

### Study design and population

An institutional-based retrospective descriptive cross-sectional study was conducted to determine the clinical profile, treatment outcomes of acute intussusception and its associated factors among children diagnosed and treated for acute intussusception at HFSUH, eastern Ethiopia. All medical records of children admitted and received management for acute intussusception at HFSUH between January 2014 and December 2020 were included in the study. Medical records of children with incomplete data and unknown treatment outcomes were excluded from the study.

### Sample size and sampling technique

All medical records of the pediatric population admitted and treated for acute intussusception between January 2014 and December 2020 at HFSUH that fulfilled the inclusion criteria were included in the study consecutively.

### Data collection tools and methods

Data were collected using a validated pretested structured data extraction checklist adopted from relevant literature and modified to the study variables. First, the operation theatre and admission records were reviewed to develop lists of cases presenting with acute intussusception between January 2014 and December 2020. Then, data were extracted from the medical registrations of children taken from the examination room at arrival, operating room records, post-surgical evaluation and monitoring sheets, and intensive care and discharge records. Data were collected by trained data collectors and supervisors through a review of the medical records of children. The variables such as socio-demographic characteristics, delay in presentation, clinical signs and symptoms, interventions given for the child, surgical procedures performed, and the duration of hospitalization were collected using the checklists.

### Interventions

The well-understood and proven interventions for children with acute intussusception were pneumatic reduction or hydrostatic enema: once intussusception is suspected and confirmed, the initial management was the pneumatic reduction, which would be attempted under mask anaesthesia or hydrostatic enema using barium enema solutions. If this attempt to a reduction of acute intussusception was unsuccessful after three trials, the reduction was considered a failure and the children would be transferred for surgical interventions.

### Data quality management

A pretested validated structured data collection tool prepared in simple English language after a review of related literature was used to ensure data quality. One day training was given to data collectors and supervisors on the purpose of the study, the contents of data collection tools, where to find the records and how to extract the required data from medical records and record data appropriately. A pretest was conducted on 5% of the sample size before the actual data collection period to check for the reliability and validity of data collection tools. The questionnaires were reviewed and checked for completeness, accuracy and consistency by the principal investigator and amended accordingly based on the pretest results. The collected data were carefully checked for incompleteness, accuracy, and inconsistency on daily basis by supervisors, and the principal investigator. Double data entry was done by two individuals to minimize errors.

### Data processing and analysis

The collected data were validated for completeness and accuracy, categorized, coded and entered into Epi-data version 3.1 and analyzed using Statistical Package for Social Sciences (SPSS) software version 25.0. Descriptive findings were expressed as frequency percentages, means, and standard deviations. The non-parametric (Chi-square (χ^2^)) tests were applied to identify the statistical significance of the association between the independent and outcome variables. The statistical significance was considered at a *p*-value < 0.05 with a 95% confidence interval (CI).

### Ethical considerations

The study was conducted following the principles of the Helsinki Declaration. Ethical clearance was obtained from the Institutional Health Research and Ethics Review Committee (IHRERC) of the College of Health and Medical Sciences, Haramaya University. A written official letter of cooperation was submitted to the HFSUH before the commencement of data collection to obtain administrative permission. Informed voluntary consent was obtained from the heads of the hospital and the department after they were informed of the aim, purpose and benefits of the study. The confidentiality of the information was maintained throughout the data collection and information dissemination process.

## Results

### Socio-demographic characteristics of study participants

In this medical records review, 86 children were admitted with the diagnoses of acute intussusception and treated at Hiwot Fana Specialized University Hospital over the seven years period, of which 75 medical records of children were included in the analysis, yielding a retrieval rate of 87.2%. Among the study participants, the majority, (72%) of children were male, giving a male to female ratio of 2.6:1. The median age of the study participant was 13 months. Regarding the age group of children, 31(44%) were in the age group of less than 12 months and 24(32%) were in the age group between 12 and 24 months, and the majority, 44(56%) of children were in the age group above one year old. The majority, 56(74.7%) of cases seen came from outside Harar (Table 1).

**Table 1:** Socio-demographic characteristics of children admitted and managed for acute intussusception at HFSUH, Eastern Ethiopia, 2021 (*n* = 75).

Variables	Frequency (*N*)	Percentage (%)
**Age of a child (in months)**		
3–6	9	12.0
7–12	24	32.0
13–24	24	32.0
25–48	12	16.0
>48	6	8.0
**Sex of a child**		
Male	54	72
Female	21	28
**Place of residence**		
Outside Harar	56	74.7
Harar	19	25.3

### Pattern of acute intussusception

**Figure 1:**
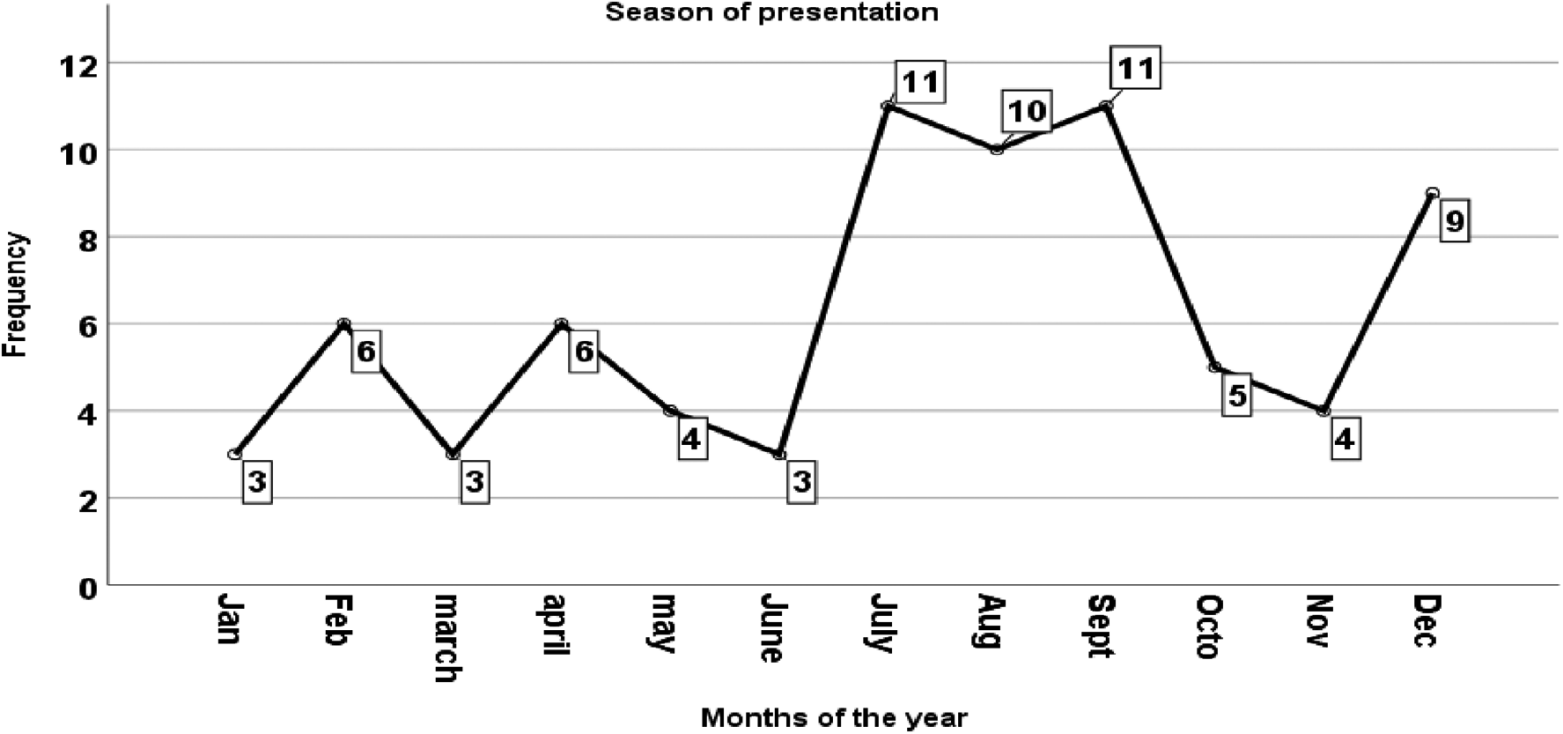
Distribution of acute intussusception presentation by months in a year among children admitted and managed for acute intussusception at HFSUH, Eastern Ethiopia, 2021 (*n* = 75).

According to the findings of this study, 32(42.7%) cases were presented between July and September, with a second peak of 10(13.3%) occurring in August (Figure 1).

### Underlining medical conditions

According to this study, only 7(9.3%) patients were identified to have signs of malnutrition, and the majority, 68(90.7%) did not have any underlying medical illness. Approximately, 40(53.3%) and 15(20%) of the children had upper respiratory tract infections and gastroenteritis within 1–2 weeks of the diagnosis of intussusception, respectively.

### Patients' clinical profile

The majority of children, 45(60%) were presented to the hospital after 48-h of the onset of symptoms, and the remaining 30(40%) were presented within 48-h of the onset of illness. Abdominal pain 71(94.7%), vomiting 70(93.3%), bloody mucoid diarrhea 53(70.7%), abdominal distention 57(76.0%), and palpable abdominal mass 24(32.0%) were the most common presenting clinical findings. The classic triads of signs and symptoms such as abdominal pain, bloody mucoid diarrhea, and abdominal mass were identified on physical examination in 32(42.7%) of cases (Table 2).

**Table 2:** Distribution of clinical presentations among children admitted and managed for acute intussusception at HFSUH, Eastern Ethiopia, 2021 (*n* = 75).

Clinical presentations	Frequency (*N*)	Percentage (%)
Abdominal pain	71	94.7
Vomiting	70	93.3
Bloody mucoid diarrhea	53	70.7
Abdominal distension	57	76.0
Abdominal mass	24	32.0
Mass protruding per anus	9	12.0
Pallor	19	25.3
Lethargy	14	18.7

### Diagnostic modalities for acute intussusception

**Figure 2:**
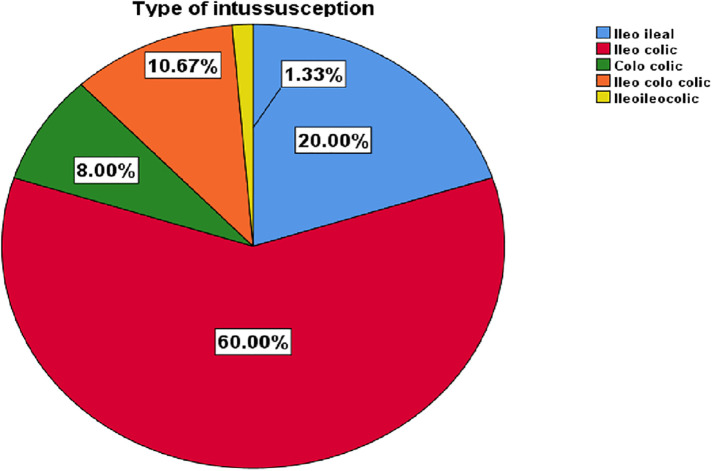
Type of acute intussusception among children admitted and managed for acute intussusception at HFSUH, Eastern Ethiopia, 2021 (*n* = 75).

Regarding the diagnostic modality, abdominal ultrasound was the most frequently used in 50(66.7%) cases followed by plain abdominal radiography in 10(13.3%), and the remaining were diagnosed clinically. Regarding the perioperative treatment, 31(41.3%) of children were treated with antibiotics and only 7(9.3%) took prophylactic antibiotics. The majority, 45(60%) of the intussusception was an ileocolic type (Figure 2). Among the total cases, 54(72%) and 4(5.3%) had non-specific lymphadenitis and Meckel's diverticulum, respectively. The remaining 17(22.7%) had no identified leading point.

### Treatment modalities for acute intussusception

In this study, all children received surgical intervention; we observed that non-surgical management options such as pneumatic reduction or hydrostatic enema services were not available in this facility. Among the surgical interventions, reduction without resection was possible in 47(62.7%) cases, resection and anastomosis were performed in 19(25.3%) cases, stoma in 2(2.7%) cases, and nothing was done for the remaining 7(9.3%) following laparotomy. Post-operative antibiotics were administered to all children who underwent surgery for acute intussusception.

### Factors associated with treatment outcomes of acute intussusception

In this study finding, the overall mortality in children with acute intussusception was 13.3% (95% CL: 11.8–14.8). Among the complications, post-operative infections and septic shock were identified as the causes of death. The majority, 65(86.7%) of children were improved and discharged with good recovery. Post-operative complications occurred in 27(36%) of patients; surgical site infection 16(21.3%), pneumonia 7(9.3%) and anastomotic leak 4(5.3%) were common complications occurring after surgical intervention. The Chi-square (*χ*^2^) test showed that there was a statistically significant association between age less than 24 months and treatment outcome of acute intussusception [*X*^2 ^= 8.13(df = 1); *p* = 0.004]. Moreover, preoperative vital signs [*X*^2 ^= 19.21(df = 2); *p* = 0.000], intraoperative findings [*X*^2 ^= 18.89 (df = 1); *p* = 0.000], and postoperative complications [*X*^2 ^= 14.60 (df = 1); *p* = 0.000] were significantly associated with treatment outcome of acute intussusception (Table 3).

**Table 3:** Factors associated with treatment outcome among children admitted and managed for acute intussusception at HFSUH, Eastern Ethiopia, 2021 (*n* = 75).

Variables	Treatment outcome				*p*-value
	Death *N* (%)	Improved *N* (%)	Subtotal *N* (%)	*χ*^2^ (df)	
**Place of residence**				1.43 (1)	0.231
Harar	1(1.3)	18(24.0)	19 (25.3%)		
Outside of Harar	9(12.0)	47(62.7)	56 (74.7)		
**Duration of hospital stay**				0.13 (1)	0.716
Less than or equal to seven days	6 (8.0)	35(46.7)	41(54.7)		
Greater than seven days	4(5.3)	30 (40.0)	34(45.3)		
**Intraoperative finding**				18.89 (1)	**0** **.** **000**
Viable	2(2.6)	52 (69.3)	54(72.0)		
Ischemic not regain viability	8(10.7)	13(17.4)	21 (28.0%)		
**Preoperative vital sign**				19.21(2)	**0** **.** **000**
Stable	1(1.3)	25 (33.3)	26 (34.7)		
Dehydrated	4 (5.3)	37 (49.3)	41 (54.7)		
Shocked	5 (6.7)	3 (4.0)	8 (10.6)		
**Duration of illness**				8.94 (1)	**0** **.** **011**
Less than or equal to 72 h	4 (5.3)	53 (70.7)	57 (76.0))		
>72-h	6 (8.0)	12 (16.0)	18 (24.0)		
**Age of a child**				8.13 (1)	**0** **.** **004**
Less than 2 years	9(12.0)	48 (64.0)	57(76.0)		
2 and above	1(1.3)	17(25.3)	18(24.0)		
**Postoperative complication**				14.60 (1)	**0** **.** **000**
No	1 (1.3)	47(62.7)	48(64.0)		
Yes	9 (12.0)	18 (24.0)	27 (36.0)		

## Discussion

Acute intussusception in pediatrics is an abdominal surgical emergency worldwide that requires timely diagnosis and interventions. Clinical case management of acute intussusception in low-income countries is different from middle and high-income countries, exclusively or predominantly depending on clinical manifestations and sometimes ultrasound for diagnosis and surgical intervention for the management (2). Strengthening collaboration with surgeons to initiate non-surgical management of intussusception is needed to reduce surgery-related complications (2). This study aimed to investigate the clinical profile, treatment outcome of acute intussusception and its associated factors among children admitted and managed for acute intussusception at Hiwot Fana Specialized University Hospital in eastern Ethiopia. In this study, the overall mortality of acute intussusception was 13.3% (95% CL: 11.8–14.8). Age < 2 years, preoperative vital signs, intraoperative findings, and postoperative complications were significantly associated with treatment outcomes among children treated for acute intussusception.

Children diagnosed with acute intussusception may manifest with varieties of signs and symptoms, which indicates the clinical importance of careful description in referral and medical records of children to facilitate timely diagnoses and interventions. The different findings indicated that the classic triad of signs and symptoms of acute intussusception such as vomiting, abdominal pain, and bloody or mucoid stools were found in 10%–66% of cases (6, 16, 17). In the present finding, the classic triads were found in 42.7% of children with acute intussusception, while the most frequent symptoms 94.7% and 93.3% were abdominal pain and vomiting, respectively. This finding was consistent with the findings of other studies (17–19).

The findings of this study indicated that the patients follow with acute intussusception was high between July and September, which is the rainy season with a second peak in December which is the dry season in Ethiopia. The present finding was similar to the findings reported in Asia (20), Africa (21), Jimma, Ethiopia (22), and Addis Ababa, Ethiopia (14). This might be explained by the higher occurrence of preceding respiratory infections and gastroenteritis in the pediatric population during the rainy and dry seasons.

In this study, the overall mortality in children with acute intussusception was 13.3%. This finding is comparable with the findings of Jimma University Medical Center (22), Tanzania (21) and systematic reviews in African countries (23). However, it was higher than the findings of studies from South Korea (24), Latin America (16), Vietnam (4), South Africa (7), Nigeria (25), and Bahrain (26). The possible reason for this discrepancy may be attributed to the huge variations in medical infrastructure accessible for the population and the quality of health care, coupled with the unavailability of recommended modern interventions for the management of acute intussusception. Moreover, associated shock on presentation, presence of gangrenous bowel intraoperatively, and postoperative complications were also noted. Delayed presentation after 48-h of the onset of symptoms, in this study, has been related to poor referral system and long distance to reach referral Hospitals in the regions. This delay in arrival to the appropriate level facility and diagnosis may also be related to low socioeconomic status and poor health-seeking behavior of the community.

The interventions for acute intussusception currently are changing from surgical intervention to non-surgical management such as hydrostatic enema and pneumatic reduction (27). However, all children in low-income countries like Ethiopia are treated with open surgical interventions, which is different from that in high-income countries. Despite non-surgical reductions having been demonstrated as being a higher rate of successful interventions for acute intussusception, poorly equipped or availability of facilities for non-surgical reductions and delayed presentations have been linked with higher morbidity and mortality in low-income countries (27–29). This finding is typically an indicator of diagnostic and interventional challenges in low-income countries. The late arrival of the child with intussusception coupled with the facilities-related challenges and lack of well-trained personnel for non-surgical reduction and poor referral systems are also reasons for poor outcomes (21, 30, 31).

As per the present findings, intraoperative findings of nonviable bowel had a significant association with treatment outcomes, and 20% of patients had gangrenous bowel intraoperatively. The present finding is in agreement with findings from Tikur Anbesa Specialized Hospital, Ethiopia (14), Jimma University Medical Center, Ethiopia (22), and Tanzania (21). However, the current finding is relatively higher than the finding from Vietnam (4). This difference is because of the late presentation of the children in this study, which has been linked with poor treatment outcomes, that is morbidity and mortality.

Available evidence indicates that delayed presentation predisposes patients to bowel complications and poor outcomes (32, 33). Similarly, the findings of this study also identified that delayed presentation is associated with poor outcomes and increased morbidity and mortality. This finding is supported by the findings of previous similar studies (13, 34).

Postoperative complications are associated with poor treatment outcomes of acute intussusception (31). In this study, 36% of the patients had postoperative complications, of which 21.3% was surgical site infection related to child mortality. This is congruent with the studies carried out in Ethiopia (33.9%) and Tanzania (42.9%) (14, 21).

### Limitations of the study

This study had some limitations: First, a retrospective document review was used, which may miss some variables and lacks explanations for why delayed presentation and timing of interventions, and due to the nature of the cross-sectional study design used, it was difficult to establish the cause-effect relationship between the study variables. Second, the study sample size was very small which may make it difficult to represent a larger population. In addition, the effect of rotavirus vaccination was not considered in this study.

## Conclusion

In this study's findings, one in seven children died of acute intussusception. Age less than 2 years, preoperative vital signs, intraoperative findings, and postoperative complications were factors significantly associated with treatment outcomes of acute intussusception. Delayed presentation of patients and lack of other treatment modalities such as non-surgical interventions are serious concerns in this facility. Therefore, attention should be given to timely diagnosis and proper referral to appropriate health facilities to improve treatment outcomes. Moreover, the initiation of pneumatic and hydrostatic reduction may reduce the requirements for surgical intervention and related complications in the long term. Furthermore, the outcomes of the child may be enhanced through improved perioperative care and reduced delays in seeking healthcare.

## Data Availability

The raw data supporting the conclusions of this article will be made available by the authors, without undue reservation.
